# Diagnostic Applications of AI in Sports: A Comprehensive Review of Injury Risk Prediction Methods

**DOI:** 10.3390/diagnostics14222516

**Published:** 2024-11-10

**Authors:** Carmina Liana Musat, Claudiu Mereuta, Aurel Nechita, Dana Tutunaru, Andreea Elena Voipan, Daniel Voipan, Elena Mereuta, Tudor Vladimir Gurau, Gabriela Gurău, Luiza Camelia Nechita

**Affiliations:** 1 Faculty of Medicine and Pharmacy, ‘Dunarea de Jos’ University of Galati, 800008 Galati, Romania; carmina.musat@ugal.ro (C.L.M.); aurel.nechita@ugal.ro (A.N.); dana.tutunaru@ugal.ro (D.T.); tudor.gurau@ugal.ro (T.V.G.); gabriela.gurau@ugal.ro (G.G.); luiza.nechita@ugal.ro (L.C.N.); 2 Faculty of Physical Education and Sport, ‘Dunarea de Jos’ University of Galati, 800008 Galati, Romania; cmereuta@ugal.ro; 3 Faculty of Automation, Computers, Electrical Engineering and Electronics, ‘Dunarea de Jos’ University of Galati, 800008 Galati, Romania; 4 Faculty of Engineering, ‘Dunarea de Jos’ University of Galati, 800008 Galati, Romania; elena.mereuta@ugal.ro

**Keywords:** injury risk prediction, artificial intelligence, sports medicine, individual sports, team sports, musculoskeletal injuries, prediction methods, technical review

## Abstract

This review provides a comprehensive analysis of the transformative role of artificial intelligence (AI) in predicting and preventing sports injuries across various disciplines. By exploring the application of machine learning (ML) and deep learning (DL) techniques, such as random forests (RFs), convolutional neural networks (CNNs), and artificial neural networks (ANNs), this review highlights AI’s ability to analyze complex datasets, detect patterns, and generate predictive insights that enhance injury prevention strategies. AI models improve the accuracy and reliability of injury risk assessments by tailoring prevention strategies to individual athlete profiles and processing real-time data. A literature review was conducted through searches in PubMed, Google Scholar, Science Direct, and Web of Science, focusing on studies from 2014 to 2024 and using keywords such as ‘artificial intelligence’, ‘machine learning’, ‘sports injury’, and ‘risk prediction’. While AI’s predictive power supports both team and individual sports, its effectiveness varies based on the unique data requirements and injury risks of each, with team sports presenting additional complexity in data integration and injury tracking across multiple players. This review also addresses critical issues such as data quality, ethical concerns, privacy, and the need for transparency in AI applications. By shifting the focus from reactive to proactive injury management, AI technologies contribute to enhanced athlete safety, optimized performance, and reduced human error in medical decisions. As AI continues to evolve, its potential to revolutionize sports injury prediction and prevention promises further advancements in athlete health and performance while addressing current challenges.

## 1. Introduction

Predicting injury risk in sports is a well-recognized goal in modern sports medicine, essential for preventing injury, optimizing athletic performance, and improving recovery strategies [1]. Research shows that by accurately predicting injury risk, personalized training and rehabilitation programs can be developed, helping athletes avoid injuries and recover more quickly when they do occur [2]. This shift from reactive treatment to preventive care is widely regarded as a significant advancement in sports medicine [3]. However, traditional methods face significant challenges, such as athlete variability, biological differences, and the complexity of modeling multiple interacting risk factors [4]. Furthermore, issues related to the quality, availability, and consistency of data, as well as reporting biases, can complicate the accuracy of injury prediction [5]. The dynamic nature of an athlete’s physiological state adds to these difficulties, making it hard for traditional models to capture real-time insights [6].

While the application of AI in healthcare has received substantial attention, its specific impact on sports injury prevention has not been fully explored. This comprehensive review consolidates the latest findings in the field, providing critical insights into how AI can transform injury prediction methods in various sports disciplines. By addressing gaps in the current literature and exploring practical applications, this review offers a foundation for future research and development in AI-driven sports medicine. In particular, ethical concerns—such as ensuring athlete data privacy and managing the secure handling of health information—are essential to address as AI technology becomes more prevalent in this field.

In recent years, AI has emerged as a promising solution to overcome the limitations of the traditional methods used in injury prediction [7]. AI systems, capable of performing tasks typically requiring human intelligence—such as learning, reasoning, and problem-solving—have already proven valuable in healthcare, especially in fields like medical imaging, diagnostics, and personalized treatment. In the context of sports, AI offers significant advantages over traditional methods by analyzing large and complex datasets to identify patterns and risk factors that might otherwise go unnoticed. Machine learning (ML), a subset of AI, has demonstrated effectiveness in predicting injury risk due to its ability to learn from historical data and refine predictions with new inputs. AI algorithms can integrate data from various sources, including wearable devices, biomechanical assessments, performance metrics, and psychological factors, creating individualized profiles for athletes [7]. By analyzing these complex, multidimensional datasets, AI can detect subtle trends or anomalies that might indicate an increased risk of injury [8]. For instance, in contact sports such as football, AI can provide real-time assessments of collision impacts, helping to predict the injury risk related to repetitive impacts and physical strain.

In addition to improving the accuracy of injury prediction, AI provides the added advantage of real-time monitoring and feedback. AI-powered systems can continuously track an athlete’s condition during training or competition, alerting coaches and medical staff to potential risks before they escalate [6]. This data-driven and personalized approach ensures that interventions are tailored to each athlete’s unique needs, enabling more effective injury prevention and management strategies [5].

Despite its promise, AI is not without its limitations. The reliability of AI models depends heavily on the quality and quantity of the data on which they are trained. Insufficient or biased data can lead to inaccurate predictions, undermining the effectiveness of AI-driven injury prevention strategies [9]. The complexity of AI models, particularly deep learning algorithms, also poses challenges for interpretation and transparency. Ethical concerns, such as privacy and the handling of sensitive health data, must be carefully considered as AI becomes more integrated into sports medicine.

A systematic review and meta-analysis were not conducted for this paper because AI in sports is a rapidly developing field, with studies using different methods and approaches across various sports. Instead, we chose a comprehensive review approach to give a broad overview of current research, covering the diverse AI techniques being used to predict injuries. This allows us to explore a wide range of studies and offer a broader understanding of AI applications in injury prevention.

This review delves into the technical aspects of the AI techniques used for injury prediction. It provides a detailed overview of ML, deep learning (DL), and generative AI (Gen-AI), and explains how these methods are applied to large and complex datasets. It also describes the search methodology used to identify relevant studies, focusing on how AI-driven models have been used for injury risk analyses in different sports.

Further analysis focuses on the use of AI in both individual and team sports. In individual sports such as athletics and alpine skiing, AI technologies are used for personalized training plans and real-time injury monitoring. These tools are adapted to meet the specific needs of each sport, with an emphasis on the continuous monitoring of performance and biomechanical data. In team sports like football, basketball, and hockey, AI is used to manage individual and team performance, monitor training loads, and predict injury risks. Various AI methods, such as ML and neural networks (NNs), are compared to evaluate their effectiveness in improving athlete health and performance. Special attention is given to musculoskeletal injuries, where AI is being used to prevent and manage injuries through the analysis of biomechanical models and clinical data, offering tailored prevention strategies for athletes.

This review concludes with a discussion of the strengths and limitations of AI in sports injury prediction, addressing ethical concerns such as data privacy and the transparency of AI models. The broader implications of AI for the future of sports medicine are considered, as well as the challenges that must be overcome to fully realize the benefits of AI-driven injury prevention.

This paper aims to analyze the role of AI in predicting sports injuries by examining key AI methods, their applications in various sports, and the challenges of implementing these technologies. It highlights both the benefits and limitations of AI in injury prevention and explores how AI can be effectively integrated into sports medicine to provide more accurate, personalized, and real-time solutions for improving athlete health and performance.

## 2. AI Methods and Searched Materials

Predicting the risk of injury in sport is a vital practice, as important for injury prevention as it is for improving athletic performance. Injury prediction is valuable for injury prevention, performance enhancement, training optimization, and rehabilitation management. However, the limitations of this method should be recognized, including individual variability, biological differences, data quality and quantity, and modeling complexity, as well as ethical issues and psychological consequences.

The accuracy and precision of the information is directly influenced by the way it is collected, reporting biases, correct medical diagnoses, and the expertise of medical professionals. Another major challenge that limits these strategies is the heterogeneity of the data and the duration of the studies.

AI is a term in computer science that refers to the creation of machines and software capable of performing functions that normally require human intelligence. These functions fall into five categories: learning, reasoning, problem solving, perception, and natural language understanding. To date, AI has mainly been applied in healthcare for AI-assisted diagnosis, medical image analysis, and the personalization of treatments. In the context of sports injury prediction, the value, limitations, accuracy, and reliability of data can be significantly improved by using AI techniques and algorithms [10,11,12,13].

This section provides an overview of the AI methods used in the studies analyzed in this review, together with a summary of the materials and datasets used to select them. The aim is to provide a clear understanding of the technical approaches and contexts in which AI methods have been used to predict injury risk in sport. Section 2.1 also aims to provide a beginner’s explanation of AI, introducing some basic concepts for readers who may not be familiar with AI. This introductory approach will cover fundamental concepts such as machine learning (ML)—the application of algorithms that learn from patters to improve predictions; deep learning (DL)—a subset of ML that uses layered neural networks to analyze complex data; and natural language processing (NLP)—the use of AI to interpret and analyze human language. The aim is to make it easier for the audience to understand how AI can be used to analyze and predict injury risk, and to ensure that those without technical expertise can follow the complexity of the studies reviewed.

### 2.1. Technical Overview of AI Methods

In the field of sports injury prediction, AI technologies are used to process complex datasets, detect patterns, and generate predictive insights. By using advanced algorithms and data analysis techniques, AI enables sports professionals to make more informed decisions, reducing the risk of injury and improving athlete performance. Various AI approaches, including ML, DL, and natural language processing (NLP) techniques [14], have been applied to injury prediction and prevention.

**Figure 1:**
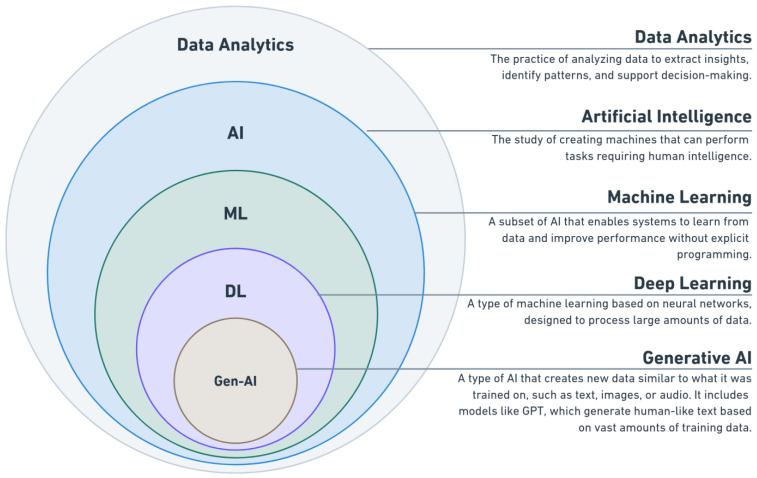
The hierarchy of the AI technologies used in sports injury prediction.

Figure 1 illustrates the relationship between different AI-related technologies, including data analytics, AI, ML, DL, and Gen-AI. Each layer in the diagram shows how these concepts fit together. These layers demonstrate the interconnection of these technologies, which all work together to provide advanced solutions for predicting and preventing injuries in sport [15].

**Figure 2:**
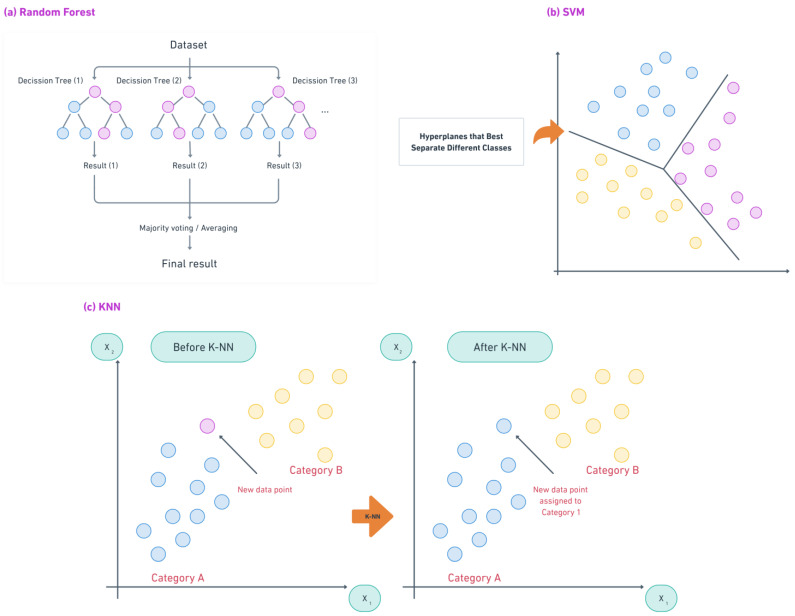
Basic architecture of ML models used in sports injury prediction: (**a**) RF; (**b**) SVM; (**c**) KNN.

**Figure 3:**
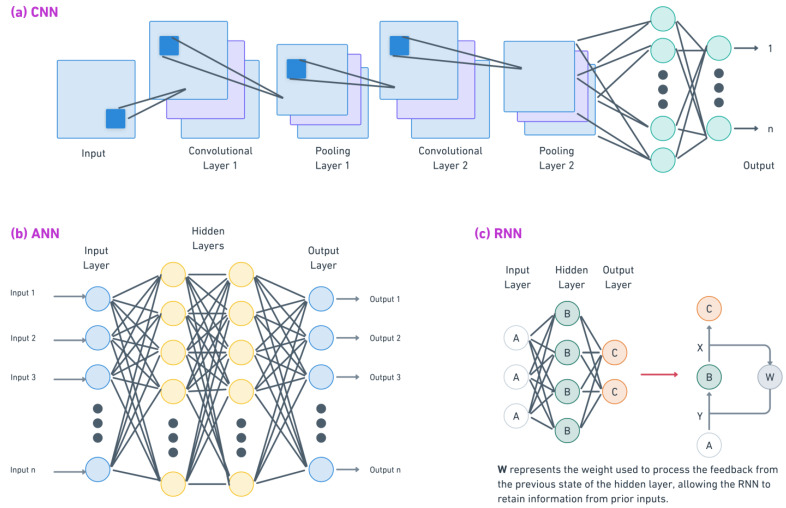
Basic architecture of DL models used in sports injury prediction: (**a**) CNN; (**b**) ANN; (**c**) RNN.

Figure 2 and Figure 3 provide a detailed representation of the underlying architectures of the main ML and DL models discussed in this paper.

#### 2.1.1. Machine Learning (ML) Techniques

At the heart of AI injury prediction is ML, which allows systems to learn from historical data and make predictions without being explicitly programmed [16]. ML models excel in sports injury prediction by identifying complex relationships between risk factors such as physical performance metrics, previous injuries, and training intensity. The main ML techniques in sports injury prediction are described below, with an emphasis on their unique features:

- Random Forests (RFs): RFs are ensemble models that create multiple decision trees and combine their results to make more accurate predictions [17]. By averaging the results of different trees, RF reduces overfitting and provides robust predictions even when dealing with complex datasets. For instance, RF models are widely used to analyze long-term data on training habits and physical performance to predict overuse injuries, particularly in endurance sports.
- Support Vector Machines (SVMs): SVMs are classifiers that work by finding an optimal boundary (hyperplane) to separate different data categories [18]. SVMs are particularly effective at distinguishing between varying levels of injury risk. For example, they can categorize athletes into high- or low-risk groups based on their training volume, biomechanical data, and recovery times, making them valuable for binary classification in injury prediction.
- K-Nearest Neighbors (KNN): KNN classifies athletes by comparing them to their closest ‘neighbors’ in the dataset, making it especially useful for grouping similar risk profiles [19]. For instance, KNN can predict muscle strain risk by comparing an athlete’s condition with similar profiles, making it a straightforward tool for practitioners.

By combining these ML techniques, sports professionals can detect complex patterns and predict injury risk with great precision. The overlapping strengths of these methods allow for their flexible application, adapting to the specific data characteristics of each sport.

#### 2.1.2. Deep Learning (DL) Techniques

DL models excel at tasks involving large and complex datasets, offering the ability to automatically learn intricate patterns without the need for manual feature extraction [20]. These techniques are particularly useful in sports injury prediction when working with data from wearables, video analysis, or sequential performance metrics. DL methods such as convolutional neural networks (CNNs), artificial neural networks (ANNs), and recurrent neural networks (RNNs) provide powerful tools for identifying subtle injury risk factors and predicting potential injuries based on an athlete’s historical and real-time data. Below are the main DL techniques used in this area:

- CNNs are deep learning models designed to analyze lattice-like data structures, such as images or video footage [21]. In sports injury prediction, CNNs are often used to analyze biomechanical data, such as an athlete’s movement patterns or video gait analysis. For example, CNNs have been used to detect abnormal running mechanics that could lead to knee injuries by processing data from wearable sensors or motion capture technology. CNNs are essential in sports that require precise motion analysis, such as athletics and gymnastics.
- ANNs consist of layers of interconnected nodes (neurons) that can model complex relationships between injury risk factors. ANNs are particularly effective at predicting a wide range of injuries, from acute muscle strains to overuse injuries, by processing variables such as training intensity, recovery time, and an athlete’s historical injury data. Their flexibility allows them to adapt to new data [22] and generate personalized injury prevention strategies, providing recommendations tailored to each athlete’s unique risk profile.
- RNNs are designed to handle sequential data, making them well suited to time series analyses in sports. RNNs excel at tasks where understanding the order and timing of data points is critical [23]. In injury prediction, RNNs can track changes in an athlete’s condition over time by analyzing sequences of performance metrics such as heart rate variability, training volume, and muscle fatigue. This ability to process temporal data makes RNNs highly effective at predicting injuries resulting from cumulative stress or overtraining.

#### 2.1.3. Generative AI

Generative Pre-trained Transformers (GPTs) are one of the most advanced AI models in natural language processing [24], and specifically GPT-4, which is based on the Transformer architecture. GPT-4 uses self-monitoring mechanisms to process and generate human-like text. While GPT-4 does not directly predict injuries, it serves as a valuable support tool in the injury prevention decision-making process by synthesizing and interpreting large volumes of text-based information. GPT-4 can synthesize large amounts of text-based data, such as medical records, injury reports, research papers, and expert opinions, to provide comprehensive summaries and insights.

For example, GPT-4 can assist medical teams by analyzing the extensive literature on specific injury types, summarizing recent studies and providing data-driven recommendations. Coaches and sports professionals can use GPT-4 to extract key insights from player health records or training logs, helping them to make more informed decisions about recovery protocols and injury prevention strategies. In practical applications, GPT-4 can analyze unstructured data, such as text reports from physiotherapists and coaches’ notes, extracting relevant details about an athlete’s condition, previous injuries, and observed patterns in recovery. By transforming these qualitative insights into actionable information, GPT-4 complements traditional data sources such as performance metrics or wearable data, contributing indirectly but meaningfully to a holistic injury prevention strategy [25].

#### 2.1.4. Data Analytics in Injury Prediction

**Figure 4:**
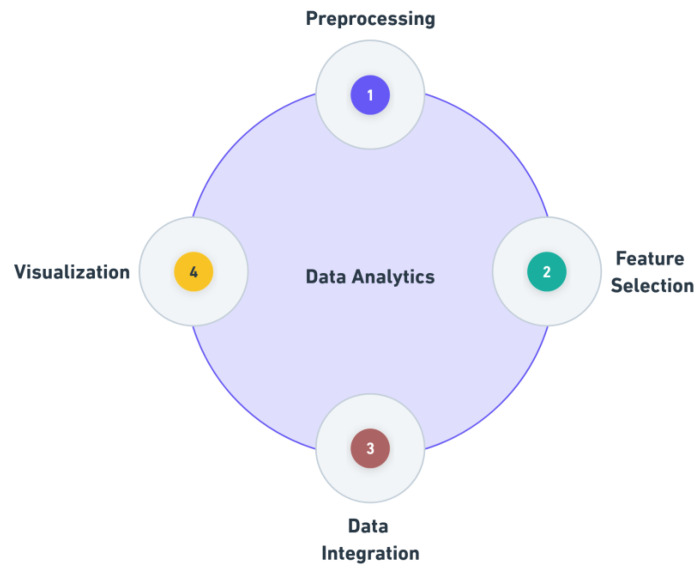
Key steps in data analysis for AI.

Data analytics is the foundation of AI applications in sports injury prediction, as illustrated in Figure 1. It involves collecting, cleaning, and interpreting data from multiple sources to make it usable for AI models [26]. The quality of the data analysis has a direct impact on the performance of AI systems, as accurate, well-structured data lead to better predictions. Figure 4 highlights the key steps in this process, which are essential for transforming raw data into a format suitable for AI-driven insights.

1. Preprocessing: This step involves standardizing data from different sources (e.g., wearable sensors, medical reports, performance logs) and dealing with missing or noisy data.
2. Feature selection: This step includes identifying the most important variables that contribute to injury risk, such as training load, previous injuries, and biomechanics, and ensuring that AI models focus on the most relevant information.
3. Data integration: This process involves combining data from multiple sources into a single dataset for analysis. In sports injury prediction, data integration is critical to bring together information from multiple systems, such as performance tracking devices, the athlete’s medical history, and environmental conditions (e.g., temperature, playing surface) [27]. Successful integration ensures that AI models have access to a comprehensive dataset that captures all relevant factors, allowing for more accurate and holistic injury risk predictions.
4. Visualization: Data visualization tools help communicate patterns and trends within the data, making it easier for coaches and sports scientists to interpret AI predictions and adjust training regimes accordingly.

By combining ML, DL, and NLP techniques, AI offers powerful tools for predicting injury risk in sports. These models not only improve early detection, but also provide personalized prevention strategies, enabling athletes to train smarter, avoid injuries, and perform at their best.

### 2.2. Search Methodology and Materials

We conducted a comprehensive review of the current literature, focusing on original articles that examined various clinical applications of AI in sports injury risk prediction. To identify relevant manuscripts, we conducted extensive searches of multiple databases, including PubMed, Google Scholar, Science Direct, and Web of Science. The search strategy was structured around three sets of keywords. The first set included the terms “artificial intelligence”, “deep learning”, and “machine learning” combined with “risk prediction”, “injury”, and “individual sport”. The second set combined the same AI-related terms with “risk prediction”, “injury”, and “team sport”. The third set focused on “artificial intelligence”, “deep learning”, and “machine learning” combined with “risk prediction”, “injury”, “sport”, and “musculoskeletal injury.”

Our search was limited to articles published in English between 2014 and 2024 to ensure that we captured the most relevant and recent studies. In addition, we consulted publications specifically related to AI in sports injury risk prediction, resulting in the identification of over 207 relevant publications. After removing duplicate articles, we conducted a detailed evaluation of the remaining articles’ abstracts and titles to assess their suitability for inclusion in the review. We identified and included a subset of 27 studies that were directly relevant to our research. These selected studies provided valuable insights into the use and impact of AI in sport and formed the basis of our review.

The selection process focused on studies that investigated the use of AI to predict injury risk in sport. We assessed each study based on several factors, including the journal of publication, the date of publication, the study design, the analytical methods used, the results, and the conclusions drawn. During the initial screening, we excluded studies that were not written in English. We also paid close attention to the quality of the data presented in each study, focusing on elements such as the rationale of the research, the design of the methods, and the results and discussions, as well as any potential evidence of methodological bias or misinterpretation of the data that could negatively affect the results of the study.

To ensure a comprehensive review, we established clear inclusion and exclusion criteria. Studies were included if they specifically examined the use of AI to predict injury risk in individual or team sports. We restricted our selection to publications in English that were published within the last 10 years (2014–2024) and published in academic journals. The exclusion criteria included articles published in languages other than English, retracted studies, short-format publications such as posters or abstracts, duplicate studies, and any studies whose titles or abstracts did not match the topic of this review.

Throughout the review process, we encountered some limitations, such as variations in the methodologies of the included studies and potential publication biases. In addition, the rapid development of AI technologies in healthcare presented a challenge in capturing the most recent research.

**Figure 5:**
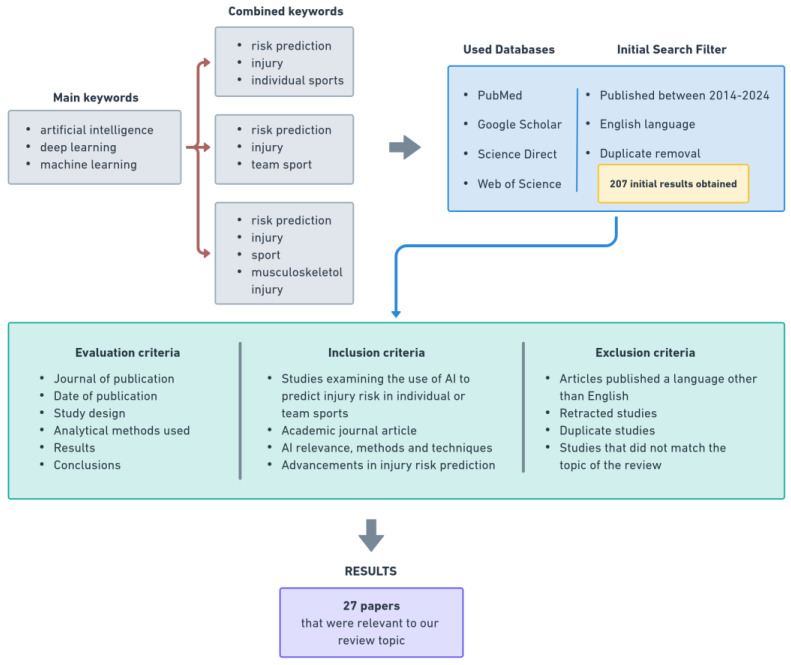
Systematic literature review process for the technical analysis of injury risk prediction methods that use AI.

To provide a clear representation of the review process, Figure 5 outlines the search flow and illustrates the stages behind the identification, selection, and inclusion of relevant studies. This figure helps to visualize the systematic review process from the initial identification of studies to the final selection of those that met the inclusion criteria.

## 3. The Role of AI in Injury Risk Prediction in Sport

Injury prevention in sport is a serious issue that needs to be prioritized for the health and performance of athletes. Injury prediction models have rapidly become proof-of-concept tools in the formulation of effective training and rehabilitation programs. Combinations of new methods and technologies, driven by advances in technology, are being used to make predictions more accurate and feasible.

However, AI is only one complementary factor that is playing an increasingly important role. For example, it helps to predict the risk of injury by analyzing large datasets, identifying patterns that could indicate potential injury and creating personalized training and recovery plans. In addition, the issue of reliability is improving as AI-driven prevention strategies introduce new, reliable options. Furthermore, using AI to prevent injuries is considered the safest and most advanced approach available. According to medical professionals, data-driven AI technologies offer significant benefits to athletes, allowing them to predict injuries and develop human-centered, personalized plans for the first time.

This section focuses on the impact of AI technology on injury risk prediction in sport, highlighting how AI supports and complements existing tools and techniques used to protect athletes and optimize their performance.

### 3.1. AI in Team Sports

In team sports like football and basketball, AI models have been used to predict muscle injuries and manage workloads. AI can also monitor collision data in rugby and American football, tracking players’ recovery from concussions and offering early warnings based on cumulative impacts. In combat sports, AI can provide predictive insights into head trauma, potentially reducing long-term health risks.

The studies [28,29] highlight AI as a proactive tool in sports medicine. AI is positioned as an early warning system that enables the identification of injury risks before they manifest. This proactive approach allows for timely intervention, shifting the focus from reactive treatment to prevention.

### 3.2. AI in Individual Sports

In individual sports like athletics, AI can monitor real-time biomechanical data, detecting signs of fatigue or poor technique that may lead to injury. In alpine skiing, AI-driven systems like mechatronic bindings adjust in real time to protect vulnerable areas like the knees. Similarly, AI can be transformative in sports such as cycling, swimming, tennis, and gymnastics, where it can analyze biomechanics to prevent overuse injuries and provide insights that improve technique and recovery.

Study [27] explores AI’s strength in real-time data processing through the use of CNNs. This technology analyzes high-dimensional data from wearable sensors, allowing AI to monitor athletes’ biomechanics and detect risky movements. Such capabilities are particularly beneficial for injury prevention in high-intensity sports, where subtle changes in movement patterns can lead to injury.

### 3.3. AI in Return-to-Play Protocols

AI also optimizes return-to-play decisions by creating personalized rehabilitation plans based on individual athletes’ data, demonstrating its ability to tailor injury management strategies. In [8], AI integrates both ML and DL techniques, highlighting its dual role as a predictive and prescriptive tool. This approach not only predicts injuries, but also provides recommendations for optimal recovery strategies. AI’s ability to both predict and guide recovery highlights its broader potential in managing an athlete’s entire rehabilitation journey.

In team sports, AI assists in tracking recovery progress post-injury, especially in contact sports where reinjury risks are higher. By monitoring recovery metrics and cumulative impacts, AI provides valuable data for determining safe return-to-play timelines, minimizing the risk of reinjury and supporting long-term athlete health.

### 3.4. Summary of AI Applications in Sports Injury Predictions

The range of AI methods applied to sports injury risk prediction is summarized in Table 1, which lists different studies and their respective AI techniques, which have been applied in different contexts.

The studies summarized in Table 1 employ a variety of AI methods, each with a unique contribution to the advancement of sports injury prediction. By comparing these roles, we gain a clearer understanding of how AI enhances injury prevention, early detection, and personalized care.

The studies [8,31] emphasize the role of AI as an analytical tool. AI analyzes different types of data to detect hidden injury patterns, while also identifying consistent injury predictors, thus contributing to the development of more accurate risk models. This analytical role is fundamental for refining our understanding of the various risk factors associated with sports injuries.

In [27], KNN serves as a simple but effective classification tool for injury risk. AI in this context provides interpretable predictions that can be easily acted upon by sports professionals. While KNN may lack the sophistication to handle highly complex datasets, its straightforward nature ensures clarity in decision-making, which is vital in time-sensitive scenarios.

Finally, ref. [33] discusses the role of GPT-4 in sports medicine. Although not directly involved in injury prediction, GPT-4 assists in making decisions by synthesizing and interpreting complex medical data. This AI application underscores the wider, indirect impact that AI can have on improving decision-making in sports medicine.

### 3.5. Broader Capabilities of AI in Injury Prediction

Beyond these specific roles, several additional insights into the broader capabilities of AI emerge from the literature. First, AI excels at processing large and complex datasets, integrating information from multiple sources such as wearable sensors, medical records, and performance logs. This comprehensive data synthesis enables sports professionals to gain a holistic view of an athlete’s condition over time. AI also improves accuracy and reduces human error in injury prediction. Traditional methods often rely on subjective judgment, whereas AI-powered models offer a more objective, data-driven approach. This results in a more accurate identification of injury risks, enabling reliable preventive measures to be taken.

AI also enables personalized injury prevention strategies. By continuously learning from new data, AI can adapt to the unique physiological profiles of individual athletes, creating real-time, dynamic care plans that evolve with each athlete’s needs.

In addition, the integration of AI into sports medicine supports real-time monitoring. AI systems can provide immediate feedback on an athlete’s biomechanics, identifying risky movements and alerting coaches before an injury occurs. This real-time monitoring is particularly valuable in elite sports, where rapid interventions can prevent serious injury.

Finally, AI has the potential to standardize injury risk prediction across different sports and training environments. By developing consistent, data-driven models, AI can set benchmarks and protocols that improve injury prevention practices globally, creating more consistent and evidence-based care for athletes across various disciplines.

Taken together, these aspects highlight the transformative potential of AI to make injury prediction more accurate, personalized, and proactive. However, realizing the full potential of AI will require addressing challenges related to data quality, model transparency, and ethical concerns. As AI technologies continue to evolve, their integration into sports medicine is likely to deepen, offering more advanced and effective solutions to improve athlete safety and performance across all sports.

## 4. The Applications of AI for Injury Risk Prediction in Sport

AI is rapidly transforming the field of sport by providing advanced methods for predicting and managing injury risk. Using ML and data analytics, AI enables more accurate and personalized assessments of athletes’ health and performance. This innovative technology is being applied to both individual and team sports (Table 2), providing tailored insights to help prevent injuries and optimize performance. This section looks at the different ways in which AI is being used in specific sports to improve athlete care and reduce the risk of injury.

### 4.1. Comparison of AI Applications in Low-Contact vs. High-Contact Sports

AI applications in injury prediction can vary significantly depending on the contact level inherent in each sport. In low-contact sports, such as tennis or athletics, AI is primarily used to monitor overuse injuries and biomechanical inefficiencies that may lead to chronic conditions like tendonitis or stress fractures. For example, AI models in tennis analyze stroke mechanics and load distribution to detect subtle asymmetries, helping to prevent injuries from repetitive motion.

In high-contact sports, such as football and rugby, AI focuses more on acute and impact-related injuries, such as concussions and ligament tears. Here, AI systems integrate real-time data from wearable sensors to assess collision intensity, monitor players’ recovery, and provide early warnings of potential reinjury. In these contexts, AI-driven monitoring has proven beneficial in reducing injury rates by tracking cumulative impact data and enabling prompt interventions, which is critical in high-contact environments.

This comparison underscores AI’s versatility in adapting injury prevention models to each sport’s unique demands, highlighting the technology’s role in both low-impact, repetitive motion injuries and high-impact, collision-related injuries.

### 4.2. Applications in Individual Sports

Individual sports provide a unique context for the implementation of AI in injury risk prediction, due to the individual characteristics of each athlete and the specific demands of each discipline. In this subsection, we will examine how AI is being used in sports such as athletics and alpine skiing, highlighting the impact of these technologies on athlete health and performance.

#### 4.2.1. Athletics

Recent studies provide interesting insights into the use of advanced technologies such as ML and AI in athletics. In a study by Rahlf et al. [34], a detailed analysis was performed using ML methods to identify biomechanical, biological, and load parameters correlated to the occurrence of injuries. This study, noted to be the first prospective longitudinal cohort study to investigate this association, has the potential to revolutionize injury prevention strategies in running by predicting injury risk and creating personalized training and recovery protocols. This approach could be extended to other sports, suggesting broad implications for athlete preparation. An important aspect of the study is the use of sensitivity analysis methods to establish a hierarchy of risk factors, helping to identify the most critical contributors to injury.

In a study by Connor et al. [35], an innovative system combining control system theory and AI techniques was introduced to create adaptive training plans that respond to unexpected changes. This system aims to improve decision-making for practitioners, enhancing both athlete development and team performance. Although focused on team sports, this methodology can be applied to other disciplines, making it a versatile tool. The study highlights that the effectiveness of these models relies on the quality of the data used, and challenges exist for practitioners who lack technical expertise in AI. Additionally, the use of personal data raises ethical and privacy concerns that must be addressed.

Both studies emphasize the importance of personalized approaches to injury risk prediction in athletics. While ref. [34] focuses on identifying and prioritizing risk factors through ML, ref. [35] highlights the adaptability of AI in managing training disruptions and improving decision-making. The role of AI in both studies is central to improving injury prevention and optimizing performance, although its specific applications differ—ref. [34] focuses on injury prediction, while ref. [35] emphasizes training adaptability.

#### 4.2.2. Alpine Skiing

In alpine skiing, AI technologies play a key role in injury prevention, especially for knee injuries—a common and serious problem in this sport. In [36], mechatronic ski bindings were developed to provide real-time adjustments based on the skier’s movements, thus reducing the risk of injury. These bindings represent a significant advance in injury prevention by providing improved protection for the knee, a particularly vulnerable area during skiing. The system can detect a skier’s position and movement, adjusting in real time to prevent injury. However, its effectiveness is limited by the lack of detailed data on skiing accidents, making calibration challenging. Continued research into injury mechanisms and integrating new technologies will be key to enhancing skier safety, potentially transforming the skiing experience by increasing safety without sacrificing enjoyment or freedom of movement.

In skiing, AI focuses on real-time injury prevention through adaptive technologies such as mechatronic ski bindings, as shown in [36]. This application contrasts with the more data-driven approaches in athletics, where AI is used to analyze large datasets to predict injuries and tailor training plans. In skiing, the role of AI is integrated into the equipment itself, providing immediate adjustments to protect the athlete, demonstrating a different but complementary application of AI in individual sports.

The applications of AI in individual sports like athletics and alpine skiing highlight the diverse ways in which AI technologies can be leveraged to enhance athlete safety and performance. In athletics, AI is used primarily for injury risk prediction and the creation of personalized training plans, while in alpine skiing, AI is integrated into equipment to provide real-time protection against injuries. These varied applications underscore the versatility of AI in addressing the unique demands of different sports, offering innovative solutions tailored to the specific needs of athletes.

### 4.3. Applications in Team Sports

In team sports, AI plays a crucial role in monitoring and analyzing both collective and individual data, providing valuable insights for injury prevention. This section explores the use of AI in various team sports, from basketball and handball to football and hockey, and highlights how these technologies are helping to keep players healthy and improve team performance.

#### 4.3.1. Basketball and Handball

In basketball and handball, AI is mainly used for performance assessment and injury risk prediction. In [37], AI is used for performance evaluation and injury risk prediction in these sports. Techniques such as ANNs model complex and non-linear relationships between variables that are critical for predicting outcomes based on a variety of input features. SVMs are used for classification tasks, effectively separating complex datasets into distinct classes, which is critical for identifying players at risk of injury. In addition, Markov processes are used to model time-dependent events such as player trajectories and match situations, enabling the dynamic analysis of performance and injury risk.

#### 4.3.2. Baseball

The application of AI in baseball has been transformative, particularly in the area of statistical analysis. In [38], AI, specifically CNNs, was used to analyze complex datasets related to player mechanics and performance. CNNs are particularly effective at handling large and complex datasets, such as pitch sequences, player swing mechanics, and fielding patterns. These networks excel at recognizing patterns in high-dimensional data, improving the accuracy of predictions related to player performance and injury risk.

The application of CNNs in baseball differs from other sports, such as basketball and handball, where AI often focuses on dynamic, real-time data. In baseball, AI is more focused on processing static images and sequences, providing detailed insights into player actions and strategies.

#### 4.3.3. Volleyball and Beach Volleyball

In volleyball and beach volleyball, AI applications focus on activity recognition and performance analysis. In [39], AI, specifically deep convolutional neural networks (deep CNNs), was used to recognize sporting actions in beach volleyball, such as spikes, blocks, and dives. The study demonstrated how deep learning models can accurately identify and analyze player movements, providing valuable feedback for injury prevention and performance improvement. This application of AI is particularly effective for real-time analysis and feedback, which are critical for injury prevention in sports where player movements are complex and varied.

Compared to the more static data analysis seen in baseball, volleyball AI applications require more dynamic and real-time processing, highlighting the adaptability of AI to different sporting contexts.

#### 4.3.4. Futsal and Football

The integration of AI into futsal and football has changed the landscape of injury risk prediction and performance management. A variety of ML techniques have been explored in multiple studies, each contributing unique insights into injury prevention and management in these sports (as summarized in Table 3).

In [42], XGBoost was used to predict injury risk in elite youth football players. The study used preseason data, including anthropometric measurements, motor coordination, and physical performance metrics, to develop a model capable of distinguishing between overuse and acute injuries. The strength of XGBoost lies in its ability to handle complex, multidimensional data, providing highly accurate predictions that are crucial for early intervention and prevention. In [41], a more comprehensive systematic review of ML applications in football was conducted. This review highlighted the widespread use of ML to analyze large datasets, particularly those generated by GPS tracking and biomechanical assessments. While the review highlighted the benefits of ML in injury prediction, it also identified challenges related to data quality and diversity, which are critical to model accuracy and reliability. In [43], a more specific application of ML was explored by focusing on neuromuscular performance parameters as predictors of non-contact injuries in elite youth football players. The study identified key neuromuscular indicators that contribute to injury risk, allowing for the development of targeted interventions. This contrasts with the broader approaches of [41,42], as ref. [43] focused on specific physiological factors for a more tailored approach to injury prevention.

In [5], a comprehensive review of ML applications in football was provided, with an emphasis on injury risk management. Unlike the previous studies, which focused on the development of specific predictive models, this review explored both the opportunities and challenges of integrating ML into routine football practice, including the need for standardized data collection and addressing ethical concerns related to predictive analytics. In [44], an innovative approach combined screening data (constant risk factors) with monitoring data (volatile risk factors) using ML. This method allows for continuous updates based on real-time data, providing a dynamic and adaptive model for injury prediction, which is particularly valuable in the fast-paced environment of professional football. In [6], the practical application of GPS training data, in conjunction with ML techniques, to predict injuries was investigated. The approach emphasized workload management using continuous monitoring to prevent overexertion, similar to the real-time monitoring seen in [44].

In [45], a biomechanical approach was taken, using ML to analyze the relationship between foot biomechanics and injury history in university football players. This study focused on biomechanical data, providing insights that could influence not only training programs, but also equipment design and rehabilitation protocols. In [46], the focus was on the prediction of hamstring injuries, a common injury in football. By using ML algorithms to analyze historical data and physiological assessments, the study provided tailored prevention strategies aimed at reducing the incidence of these injuries. In [47], KNN and ANNs were compared for the prediction of muscle injuries through biomechanical analysis. This study highlighted the strengths of both methods in providing accurate and explainable predictions, with KNN struggling with high-dimensional data and ANN requiring large datasets.

Across these studies, ML techniques are being applied in diverse and complementary ways to address the complex challenges of injury prevention in football. While refs. [40,42] focus on specific physiological and performance metrics and offer targeted intervention strategies, refs. [41,43] provide broader overviews, emphasizing the integration of ML into wider football practices. Both refs. [5,44] use real-time data, but ref. [5] takes a more dynamic and adaptive approach, while ref. [44] emphasizes workload management to prevent overuse injuries.

The biomechanical focus in [6] adds another dimension to injury prediction, providing structural insights that could influence both prevention and equipment design. Refs. [45,46] narrow the focus to specific injury types and methodological comparisons, providing deeper insights into the factors leading to injury and the effectiveness of different ML approaches.

Based on the reviewed studies, XGBoost, as applied in [42], appears to be the most effective method for predicting injury risk in football. XGBoost’s ability to handle complex and multidimensional data with high accuracy makes it well suited for early injury prediction and prevention. Its flexibility in dealing with large datasets and its proven accuracy in distinguishing between different types of injuries, particularly in youth football players, make it a valuable tool for real-time sports management. However, in terms of real-time adaptability, the approach in [44], which combines static and dynamic data, also stands out for its ability to continuously update and respond to changing conditions during training and matches.

These studies illustrate the versatility and depth of AI applications in football. Each technique offers unique benefits depending on its specific objectives—whether it is managing training loads, predicting specific types of injury, or optimizing real-time decision-making. Together, they highlight the growing importance of AI in improving both injury prevention and overall performance in football.

#### 4.3.5. Hockey

In hockey, AI applications have focused on the development of advanced injury prediction models using modern processing technologies and ML architectures. In [48], ML models, including RF and neural networks, were shown to outperform traditional logistic regression methods in predicting injury risks for National Hockey League (NHL) players. The study highlighted how these advanced models can analyze a wide range of data—from performance metrics and historical injury records to game conditions—to provide more accurate and dynamic predictions.

One of the key strengths of these ML models is their ability to continuously update predictions as new data become available, making them particularly effective in a fast-paced sport like hockey, where risk factors can change rapidly. This application of AI in hockey highlights the importance of adaptive, data-driven approaches to player health management and injury prevention in high-stakes sporting environments.

In team sports, the application of AI has proven to be an essential tool in improving both injury prevention and performance management. In basketball, handball, baseball, volleyball, football, futsal, and hockey, AI techniques such as ANNs, CNNs, KNN, and RFs have been instrumental in analyzing large datasets and providing real-time, dynamic predictions.

In sports such as basketball and handball, AI helps model the complex relationships between variables, providing valuable insights for performance evaluation and injury risk prediction. In baseball, AI focuses on processing static data such as pitch sequences and swing mechanics, improving the accuracy of predicting player performance and injury risk. In volleyball and beach volleyball, AI’s real-time activity detection and motion analysis have contributed significantly to injury prevention and performance enhancement.

Football and futsal have seen some of the most diverse applications of AI, with techniques ranging from XGBoost to neural networks. These tools not only predict injuries, but also offer data-driven strategies to manage training loads and reduce overexertion. Hockey’s fast-paced environment benefits from adaptive AI models such as RF and neural networks (NNs) [49], which continually update predictions based on new data.

In these team sports, the role of AI is multifaceted—helping to monitor both collective and individual player data, managing workloads, and predicting injury risk with increasing accuracy. AI’s ability to integrate real-time data into decision-making processes has revolutionized the way teams approach injury prevention, making it an indispensable part of modern sports management.

Ultimately, AI in team sports is driving a shift from reactive injury management to proactive injury prevention, ensuring better health outcomes and improving overall team performance.

### 4.4. Applications in Musculoskeletal Injury

Musculoskeletal injuries are a major problem in sport, affecting both professional and amateur athletes. AI offers advanced solutions for predicting and preventing these types of injuries through the use of algorithms that analyze biomechanical models and clinical data. In this subsection, we will explore the various applications of AI in the prevention and management of musculoskeletal injuries, highlighting its effectiveness in reducing the risk of injury.

Two key studies, summarized in Table 4, discuss the use of advanced technologies and predictive models in sport to manage and prevent musculoskeletal injuries, particularly those affecting the lower limbs.

In [50], the focus was on using ML to predict the risk of lower-extremity musculoskeletal injuries in student athletes. The researchers used a RF model to analyze various physical performance metrics, including joint strength, postural stability, flexibility, previous injury history, and demographics. The RF model achieved 79% accuracy in predicting injury risk, identifying significant risk factors such as hip adductor strength, hip external rotation strength, and flexibility measures such as straight-leg raises. The authors emphasized the importance of these factors in injury prevention, suggesting that the early identification of high-risk individuals could allow for timely interventions to reduce the occurrence of chronic or career-ending injuries. In addition, the study was developed a web-based application to facilitate communication between athletes, coaches, and clinicians, thereby enhancing the practical application of the findings.

In [51], researchers explored the use of advanced statistical methods from ML and data mining to build robust predictive models for muscle injuries, especially in the lower limbs. The study compared several ML techniques to identify athletes at high risk of muscle injury, focusing on football and handball players. The models demonstrated moderate accuracy in identifying injury risk, highlighting the importance of integrating biomechanical and demographic data to improve prediction outcomes. The authors also found that the quality and quantity of data had a significant impact on the effectiveness of these models, and that such predictive tools could aid strategic decision-making for training and health management.

Both studies highlight the crucial role of AI in improving the prediction of musculoskeletal injuries, although they focus on different aspects. In [50], the authors emphasize the practical application of ML in real-world settings, with the development of a web-based application to support injury prevention. Meanwhile, ref. [51] took a more technical approach, comparing different ML techniques to identify the most effective methods for injury prediction. Despite these differences, both studies highlight the potential of AI to improve injury prevention strategies by providing early warning signs and enabling personalized interventions.

The application of AI in the prediction of musculoskeletal injury risk has shown promising results in both research and practical settings. By using ML models that incorporate a wide range of biomechanical and demographic data, these studies have demonstrated the potential for AI to significantly reduce the incidence of injury among athletes. The development of practical tools, such as web applications, further enhances the usability of these models, making them accessible to coaches, trainers, and medical professionals. As AI technology continues to advance, its role in sports injury prevention is likely to become increasingly integral, providing more precise and personalized solutions for athlete health and performance.

## 5. Discussion

The integration of AI into sports injury risk prediction offers us opportunities to improve athlete care by analyzing large, complex datasets and providing personalized, real-time insights. AI systems can identify injury risks and provide proactive solutions for improving performance, preventing injuries, and aiding rehabilitation by analyzing data from wearables, performance metrics, and biomechanical assessments.

In individual sports like athletics, AI can monitor real-time biomechanical data, detecting signs of fatigue or poor technique that may lead to injury. In alpine skiing, AI-driven systems like mechatronic bindings adjust in real time to protect vulnerable areas like the knees. Similarly, AI can be transformative in sports such as cycling, swimming, tennis, and gymnastics, where it can analyze biomechanics to prevent overuse injuries and provide insights to improve technique and recovery.

AI also plays a key role in managing musculoskeletal injuries and predicting strains, tears, and joint injuries by analyzing biomechanical data and muscle fatigue in real time. By offering early intervention strategies, AI can help prevent injuries before they occur.

Despite its benefits, AI faces challenges related to data quality, model interpretability, and ethical concerns. The accuracy of AI predictions depends on the quality and completeness of the data, and the complexity of AI models can make them difficult for practitioners to interpret. Ethical concerns, particularly around data privacy, also require careful attention. This review is also limited by the fact that it is not a systematic review or meta-analysis, which may limit the generalization of its findings. Moreover, the studies analyzed vary widely in their methodologies and sample sizes, which could affect the consistency and applicability of the results. AI’s reliance on historical data also presents limitations, as these systems can be less effective when faced with new or incomplete data. Another limitation is the rapid pace of AI advancements, which may render some of the reviewed studies outdated or less relevant in the near future.

As AI technology continues to evolve, many of these challenges are expected to be addressed, further enhancing its role in sports injury prevention. The expansion of AI across different sports will lead to safer training environments and better protection against preventable injuries, marking a significant advancement in sports medicine.

### Ethical Considerations and Future Directions

As AI becomes increasingly integrated into sports injury prevention, ethical considerations play a critical role in ensuring the responsible and transparent use of this technology. Issues around data privacy, model interpretability, and responsible data handling are fundamental to maintaining athlete trust and adherence to regulatory standards. In the context of sports medicine, frameworks such as the General Data Protection Regulation (GDPR) in Europe and the Health Insurance Portability and Accountability Act (HIPAA) in the United States offer guidelines for managing personal health data, protecting athletes’ privacy, and ensuring informed consent. These regulations help shape AI applications in sports by requiring data minimization, anonymization, and controlled access, particularly when dealing with sensitive medical and performance data.

For example, under GDPR, personal health data from wearables and medical assessments must be anonymized or pseudonymized to prevent unauthorized access. Compliance with GDPR and HIPAA principles also entails implementing clear data access protocols and ensuring that data are only used for their intended purposes, such as injury prevention, performance enhancement, or rehabilitation.

Transparency in AI models is another critical area that requires further development. Given the complexity of some AI algorithms, particularly deep learning models, there is a growing need for interpretable AI solutions in sports. Transparent models can help medical teams and coaches understand the rationale behind AI predictions, thereby facilitating more informed decision-making. Methods such as explainable AI (XAI) tools, which clarify the decision pathways of AI systems, can enhance model transparency and foster trust among stakeholders.

Future directions for AI in sports medicine will likely involve establishing specialized ethics guidelines specific to sports contexts, potentially integrating sports medicine councils or athlete associations to create best practices for data handling and model deployment. Additionally, as AI technologies advance, ongoing updates to ethical frameworks will be necessary to address emerging challenges, such as new data sources (e.g., biometric data from wearables) and evolving data analysis techniques.

In conclusion, a balanced approach to AI integration in sports medicine—one that respects privacy, ensures transparency, and leverages data responsibly—will be crucial for achieving sustainable, ethical advancements in athlete care.

## 6. Conclusions

The integration of AI into sports injury prediction offers us significant opportunities to enhance athlete care by analyzing complex datasets and providing personalized, real-time insights. In individual sports like athletics, alpine skiing, cycling, and swimming, AI monitors biomechanical data to detect fatigue and inefficiencies, enabling dynamic adjustments and tailored injury prevention strategies. Similarly, in team sports such as rugby, soccer, and basketball, AI helps manage workload, track recovery, and prevent overexertion by analyzing real-time data such as collision impacts and fatigue levels.

AI is particularly effective at managing musculoskeletal injuries as it predicts and prevents injuries through the real-time analysis of biomechanical models, muscle fatigue, and joint stability. However, challenges remain regarding data quality, model interpretability, and ethical concerns related to data privacy and transparency.

Future research should focus on conducting longitudinal studies to better understand the long-term effects of AI on injury prevention and athlete health across diverse sports disciplines. Validation studies in different sports environments and among various athlete demographics will be essential to improve the accuracy and generalizability of AI models. Additionally, collaborative research between AI developers and sports medicine professionals could help address ethical and interpretability challenges, ensuring that AI advancements remain aligned with athlete safety and privacy standards.

As AI technology evolves, improvements in data quality and model transparency are expected to enhance injury prevention strategies, providing athletes with safer training environments and better protection from preventable injuries.

## Figures and Tables

**Table 1:** Papers analyzing the role of AI in sports injury risk prediction.

AI Method	Author (Year)	Application
ML	Ayala RED (2024) [28]	Early identification of injury risks in athletes using ML techniques.
	Desai V. (2024) [29]	Future of AI in sports medicine and return-to-play scenarios.
	Van Eetvelde H.(2021) [8]	Systematic review of ML methods in the prediction and prevention of sports injuries.
ML, DL	Ramkumar PN(2022) [30]	Use of AI in sports medicine, focusing on injury prediction and recovery.
RF, L1	Jauhiainen S.(2021) [31]	Detection of injury risk factors in young athletes.
KNN	Amendolara A.(2023) [27]	Overview of ML applications in sports injury prediction.
CNN	Link J. (2022) [32]	Activity recognition in ultimate frisbee using wearable sensors.
GPT-4	Cheng K. (2023) [33]	Application of GPT-4 in sports medicine, exploring AI’s role in decision-making.

**Table 2:** Sport and injury metrics addressed by AI techniques.

Sport	AI Method	Author (Year)	Application	Sample Size	Data Source
Athletics	ML	Rahlf A.L. (2022) [34]	Identification of risk factors for running-related injuries using ML techniques.	120 runners	Private, collected via surveys and biomechanical measurements
		Connor M. (2022) [35]	Adaptive training plan generation using control systems and AI.	Simulation (N = 1800)	Simulated data, no real data collected
Alpine Skiing	ML	Hermann A. (2021) [36]	Knee injury prevention in alpine skiing using mechatronic ski bindings.	Not specified	Research-based, design, and biomechanical assessment
Basketball Handball	ML (ANN, SVM, Markov ^1^)	Claudino J.G (2019) [37]	Evaluation of performance and injury risk in basketball, handball, and volleyball.	6456 athletes (various sports)	Public, data from academic research
Baseball	CNN	Koseler K. (2017) [38]	Statistical analysis and injury risk prediction in baseball.	Not specified	Public, systematic literature review
Volleyball Beach Volleyball	Deep CNN	Kautz T. (2017) [39]	Activity recognition and performance analysis in beach volleyball.	30 participants (11 women and 19 men)	Private, collected via wearable sensors attached to the wrist
Futsal	RF, XGBoost ^2^	Ruiz-Pérez I. (2021) [40]	Predicting lower-extremity soft tissue injuries in elite futsal players.	139 players	Private, preseason screening and monitoring
Football	ML	Rico-González M. (2023) [41]	Focused on a common injury type.	Not specified	Public, systematic literature review
	ML (XGBoost)	Rommers N. (2020) [42]	Systematic review of ML applications in football for injury risk prediction.	734 players	Private, data from football academy monitoring
	ML	Kolodziej M. (2021) [43]	Injury risk assessment in elite youth football players.	62 players	Private, collected from neuromuscular performance tests
		Nassis GP. (2023) [5]	Identification of neuromuscular performance parameters as risk factors for non-contact injuries.	Not specified	Public, literature Review
		Hecksteden A. (2023) [44]	Review of ML applications in football with an emphasis on injury risk.	84 players	Private, screening and monitoring with ML methods
		Rossi A. (2018) [6]	Combining screening and monitoring data with ML for injury prediction.	Not specified	Private, GPS data from training sessions and games
		Windsor J. (2022) [45]	Effective injury forecasting in football using GPS training data and ML techniques.	77 players	Private, biomechanical assessments and medical history
		Ayala F. (2019) [46]	Analysis of foot biomechanics and injury history in varsity football athletes.	96 players	Private, preseason screening
	KNN, ANN	Calderón-Díaz M. (2023) [47]	Development of preventive models for hamstring injuries in professional soccer.	Not specified	Private, biomechanical analysis with sensors
Hockey	RF, Neural Networks	Schickendantz M.S. (2020) [48]	Injury risk prediction and management in NHL hockey players.	2322 players	Public, data from publicly reported NHL injury databases

^1^ A Markov model is a type of statistical model that predicts future states based only on the current state, without requiring knowledge of the sequence of events that preceded it. ^2^ Extreme Gradient Boosting is a powerful ML algorithm based on decision trees. It builds multiple trees sequentially, with each new tree correcting errors from the previous ones.

**Table 3:** Overview of ML techniques applied in futsal and football injury risk prediction and performance management.

Study	Methods Used	Key Findings	Pros	Cons	Applications
Rommers (2020) [42]	XGBoost	Predicts overuse and acute injuries in youth football players.	Highly accurate for complex data.	Needs large amount of high-quality data.	Early injury prediction.
Rico-González (2023) [41]	Review (various ML techniques)	Highlights trends in using ML for injury prediction.	Broad overview of ML in football.	Relies on existing research; no new data.	Guides future research.
Kolodziej (2021) [43]	ML (specific model not specified)	Identifies neuromuscular risks for non-contact injuries.	Focuses on specific risk factors.	Limited to neuromuscular data.	Targeted injury prevention.
Nassis GP (2023) [5]	Review (various ML techniques)	Discusses ML’s role and challenges in football injury management.	Addresses practical and ethical issues.	Theoretical; lacks new data.	Guides ethical use of ML in football.
Hecksteden A. (2023) [44]	Combined ML (screening and monitoring)	Combines static and dynamic data for real-time predictions.	Adaptable and accurate in real time.	Complex to implement.	Real-time injury management.
Rossi A. (2018) [6]	ML (GPS data analysis)	Uses GPS data to predict injuries and manage workload.	Practical, helps prevent overexertion.	GPS data might miss some risk factors.	Workload management.
Windsor (2022) [45]	ML (foot biomechanics)	Links foot biomechanics to injury history.	Offers specific, actionable insights.	Focuses only on biomechanical data.	Customized training and rehabilitation.
Ayala F. (2019) [46]	ML (preventive models)	Predicts hamstring injuries in soccer players.	Focuses on a common injury type.	Limited to hamstring injuries.	Hamstring injury prevention.
Calderón-Díaz (2023) [47]	KNN, ANN	Compares KNN and ANN for predicting muscle injuries.	Accurate and explainable predictions.	KNN struggles with high-dimensional data; ANN needs large datasets.	Muscle injury prediction.

**Table 4:** AI applications for predicting musculoskeletal injury risk.

AI Method	Author (Year)	Application
RF	Henriquez M. (2020) [50]	Prediction of lower-extremity musculoskeletal injury risk in student athletes using an RF model.
ML, Data Mining	López-Valenciano A. (2018) [51]	Development of predictive models for muscle injuries in football and handball players using ML techniques.

## Data Availability

Not applicable.

## Abbreviations

AI	Artificial Intelligence
ANN	Artificial Neural Network
CNN	Convolutional Neural Network
DL	Deep Learning
Gen-AI	Generative AI
GPT	Generative Pre-trained Transformer
KNN	K-Nearest Neighbors
L1	Lasso Regression (a type of regularization method)
ML	Machine Learning
NHL	National Hockey League
NLP	Natural Language Processing
RF	Random Forest
RNN	Recurrent Neural Network
SVM	Support Vector Machine
XGBoost	Extreme Gradient Boosting
